# How to pick a winning team: approaches towards the selection of computationally derived protein structures for ensemble-based virtual screening

**DOI:** 10.1186/1758-2946-5-S1-O7

**Published:** 2013-03-22

**Authors:** Barbara Sander, Oliver Korb, Jason Cole, Jonathan W Essex

**Affiliations:** 1 School of Chemistry, University of Southampton, Highfield, Southampton, SO17 1BJ, UK; 2 Cambridge Crystallographic Data Centre, 12 Union Road, Cambridge, CB2 1EZ, UK

## 

The necessity of treating receptor flexibility in protein-ligand docking has been widely acknowledged and is the subject of extensive research in the field of drug discovery [1]. The use of multiple discrete protein conformations, so-called ensemble docking, has been proven to be a valid concept to mimic target plasticity in docking experiments [2,3]. Using molecular dynamics (MD) the number of different conformations that can be generated is practically unlimited. Not all of these conformations can be included in the pose prediction or virtual screening process for reasons of computational cost. Moreover, some of them will be more suitable for docking purposes than others. The question arises if and how adequate protein conformations can be selected systematically a priori based on quantifiable structural features.

For neuraminidase and cyclin-dependent kinase II, snapshots of molecular dynamics simulation trajectories have been clustered and structurally assessed by applying a variety of methods. Extensive cross docking and virtual screening experiments show that relatively large differences in docking performance are caused by only very subtle conformational changes within the protein which cannot be captured by the currently applied characterisation methods. As an alternative, cross docking capability can be used as a reliable indicator towards the selection of suitable conformations for ensemble-based virtual screening. In combination with short minimisations of docked poses in the binding site, virtual screening performance can be further improved and ensembles of MD snapshots can be built which perform as well as the generally superior holo crystal structures.
